# Effectiveness of Occupational Safety and Health interventions: a long way to go

**DOI:** 10.3389/fpubh.2024.1292692

**Published:** 2024-05-09

**Authors:** Gaia Vitrano, Guido J. L. Micheli

**Affiliations:** Department of Management, Economics and Industrial Engineering, Politecnico di Milano, Milan, Italy

**Keywords:** literature review, integrative review, interventions, effectiveness, occupational health, occupational safety, management, research agenda

## Abstract

**Background:**

Occupational Safety and Health (OSH) has become an area of increasing concern for organizations and institutions. As it evolves, it has gradually posed ongoing challenges, becoming more complex, for organizations. Consequently, more comprehensive studies are required to advance academic and institutional research. From this perspective, this study aims to gather research contributions on the effectiveness of existing interventions for OSH improvement and identify areas for further exploration.

**Methods:**

According to the nature of scientific literature, the overall process of a literature review was investigated following an integrative approach, which involved searching for, selecting, and analyzing various literature in a creative and integrated manner, without a predefined structure.

**Results:**

The analysis suggests that there is room for improvement in understanding the effectiveness of OSH interventions and more concrete guidance is still desirable. Based on the literature, some research areas for future developments in OSH interventions are identified. One potential area to explore further is fostering human-centered technological development and a more conscious network of stakeholders, with higher coordination, shared knowledge, and open communication.

**Implications:**

Focusing on the proposed directions will support scholars and practitioners in pursuing continuous OSH improvement through more effective and well-grounded workplace interventions and encourage organizations to be proactive in daily OSH management.

## 1 Introduction: a practical issue

Considering the international statistics on occupational accidents and diseases, an alarming situation with an increasing trend is evident. Recently, the International Labor Organization (ILO) (1) estimated the annual global work deaths to be 2.78 million, ~7,600 per day (2). Work-related deaths in Asia account for two-thirds of the total global workplace fatalities, whereas those in Africa and Europe account for < 12% (3). ILO calculated approximately 340 million occupational accidents worldwide and 160 million victims of work-related diseases annually, with an increasing trend (4). The corresponding loss of workdays accounts for US $3.2 trillion, comparable to nearly 4% of the global GDP (3). Workplace health and safety management and promotion may positively impact workers and leadership and engagement at all levels are key issues in changing the workplace culture (5).

In this context, effective Occupational Safety and Health (OSH) interventions are a leading priority, particularly for organizations struggling to manage health and safety in the workplace (6, 7). OSH is a discipline focusing on the prevention of work-related injuries and diseases and the promotion of the health, safety, and wellbeing of the workers at the workplace by improving their working conditions (8). Advancing research on OSH toward more theoretical and strategic perspectives and investigating how to constantly improve OSH management at the system level could enhance OSH interventions on the ground (9). There is, in this respect, a growing interest in OSH management performance among public institutions, which are allocating considerable resources toward improving workplace OSH conditions (10–12); however, it is essential, and more studies are still needed, to assess the effectiveness of these efforts (13).

In the last years, scholars have emphasized that assessing the effectiveness of interventions is crucial for maximizing their impact and working for their continuous improvement (13). Nonetheless, their effectiveness is still rarely monitored and often assumed without proper assessment since considered too difficult to measure as interventions often operate in nuanced contexts, relying on myriad qualitative factors that are difficult to track (14–16). Hence, a discussion has been introduced in the literature on the effectiveness of OSH interventions, however, a comprehensive view of the overall problem is still not plain and understanding the status quo and identifying potential improvement areas will make scholars and practitioners aware of the major issues and will support them in pursuing higher effectiveness in OSH interventions.

In this regard, through a review of the OSH literature, this study aimed to gather research contributions on the effectiveness of existing interventions, derive knowledge on how researchers are moving forward toward more effective interventions for OSH improvement and identify areas that merit deeper exploration.

According to the nature of scientific literature, the overall literature review process has been investigated following an integrative approach (17), which involves searching for, selecting, and analyzing various literature in a creative and integrated manner, without a predefined structure. This allows researchers to provide a comprehensive understanding of complex concepts while not aiming to include all published work on the topic, which would potentially turn into an endless process, but rather to consistently pursue the research objective by combining different perspectives and obtaining relevant findings. Accordingly, this study examined a specific branch of literature that investigated the effectiveness of interventions from different perspectives, and options for their improvement without intentionally including all extant literature on OSH interventions, which is beyond the scope of this study.

## 2 Methods: literature review process

To examine the current state of interventions for OSH improvement, this study reviewed OSH literature, following Snyder's (17) integrative approach. Different types of literature reviews exist; according to Snyder (17), they can be classified as purely systematic, semi-systematic, or integrative reviews. A “best option” does not exist, and the choice depends on the field and scope of the study. This study adopted an integrative approach (18). According to Torraco (18), an integrative literature review is a sophisticated form of research that requires a great deal of research skill and insight and is not less rigorous than other types of research. An integrative literature review is a form of research that searches for, selects, and analyzes documents in an integrated manner (18), which implies that there is no canonical structure to follow; it is shaped by the research itself.

Since exhaustiveness for literature selection is outside of the scope, or simply not possible, in integrative literature reviews, authors are expected to justify the selection of included literature and analyze and critique the literature by applying techniques that are not set in advance, since there is no well-established format to organize collected articles (18).

Integrative literature reviews are suggested to address both mature and newly emerging topics and strategies for searching and reviewing documents change according to the maturity of the addressed topic. The OSH field might be considered a mature topic, although the literature is less structured and quite dispersed, with high research potential. In this case, an integrative approach can grasp different facets of the OSH literature and more sufficiently answer the research objective.

Although an integrative literature review article can be organized in various ways, it is expected to follow a process that includes the literature search, selection, analysis, and critical synthesis. Regarding other review types, readers of an integrative literature review expect transparency concerning the review process, that is, how the findings of the study are obtained (18). Integrative literature reviews combine different search processes, which do not prevent researchers from performing systematic searches; instead, they provide the chance to perform more than one systematic search complemented by other sources derived from a snowballing process. Therefore, a single systematic search would not be exhaustive and might ignore relevant sources; hence, an integrative literature search provides added value.

### 2.1 The search process

**Figure 1:**
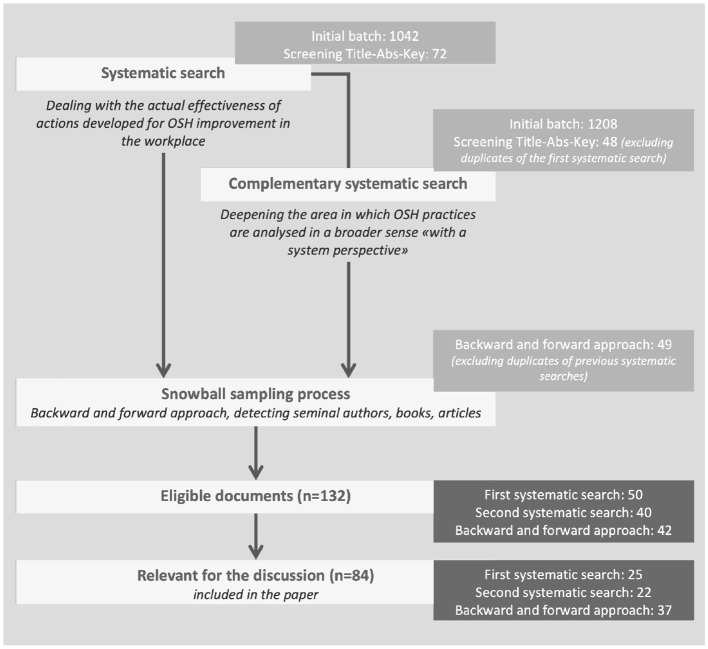
The search process.

During the search process, two main systematic searches were applied to investigate the OSH literature from theoretical and practical perspectives. This supported the subsequent snowball sampling process until the final eligible documents for review were identified. Out of the 132 pertinent documents, 84 were considered more significant, and explicitly included in the discussion. The search process phases are illustrated in Figure 1.

The main search protocol in the Scopus database was developed to deepen the core themes of this study and identify possible seminal documents. It aimed to locate documents in the OSH field dealing with the actual effectiveness of interventions developed to improve OSH in the workplace. It was divided into three major blocks:

- The context: OSH.
- The area of application: interventions, and synonyms.
- The aim: performance, outcome, and synonyms.

The resulting query was TITLE-ABS-KEY ([“occupational” W/3 “health” W/3 “safety” OR “OSH” OR “OHS” AND “occupational” AND “health” AND “safety”] W/4 [“intervention^*^” OR “initiative^*^” OR “program^*^” OR “instrument^*^” OR “project^*^” OR “measure^*^” OR “practice^*^”] AND [“performance^*^” OR “effect^*^” OR “effic^*^” OR “indicator^*^” OR “outcome^*^” OR “output^*^” OR “impact^*^”]). A total of 1,042 documents were identified.

The choice of keywords and all potential synonyms was based on the Authors' previous knowledge of the topic and was complemented by reading the keywords applied in a recent EU-OSHA report for the European project SESAME (19). This project was developed in collaboration with nine EU Member States and identified effective programs at the operational and policy level that could lead to improvements in OSH in Micro and Small Enterprises (MSEs), by defining “what works, for whom, and in what circumstances” (20). The operator *W/4* (within 4) was used instead of *AND* because the selected documents should only refer to OSH interventions (or synonyms) and not to general ones developed in the OSH field. However, it was not possible to precisely quantify the maximum distance between the words “intervention” and “performance”, and the operator *AND* was applied.

By reading documents, it seemed that studies with a system view of OSH matters showed higher effectiveness in OSH interventions; therefore, another complementary search protocol was performed in the Scopus database to examine a specific cluster of documents. The resulting query was TITLE-ABS-KEY ([“occupational” W/3 “health” W/3 “safety”] OR [“OSH”] OR [“OHS”] AND [“occupational” AND “health” AND “safety”] W/3 [“network^*^” OR “system^*^” OR “framework^*^”]). A total of 1,208 documents were identified.

Once the first batch of documents was identified, other documents were selected following both backward and forward approaches by examining the cited studies of the selected documents (Figure 1). Both authors employed these approaches to integrate additional documents into the analysis. Consensus was achieved through a comparison of newly included documents by both authors, and any discrepancies were reviewed together to determine their inclusion or exclusion.

This process was guided by co-citation analysis conducted using the VOSviewer software, which is open-source software used to visualize and analyze networks that display connections between different elements, visualizing clusters of similar elements, i.e., relationships between authors, concepts, or topics within a corpus of texts. In particular, co-citation analysis identifies connections between documents, authors, or journals based on their co-citation patterns. This analysis facilitated the tracing of seminal studies and connections between different areas of study. However, despite its advantages, co-citation analysis relies on cited articles and citations take time to accumulate, making it challenging to relate new publications directly to existing literature. For this reason, a forward approach, which involves identifying recent documents citing seminal studies identified through co-citation analysis, was considered crucial to also include new relevant publications.

The search and selection processes were considered reasonably exhaustive when documents almost converged, that is when selected studies showed a significant number of commonly cited sources.

### 2.2 Data analysis

Among the relevant studies for analysis, a document reduction was performed by reading the abstracts, titles, and keywords and eliminating those that were outside of the scope. The final batch of documents to be included was determined by reading the full studies of the selected abstracts. Both authors meticulously reviewed the documents, collaborating to identify the final set of studies for inclusion. Specifically, one author primarily undertook the task of reviewing documents from the two systematic searches and the snowballing sampling process, while the other mostly supervised the whole process, defining the set of documents for analysis.

To ensure a quality data analysis process, documents were analyzed and coded into a data form that included the normal identification data and the core literature review data, which was selected by reading the studies. This approach facilitated the process of comparing primary sources because, owing to the built data extraction form, documents were reduced to a single-page format with similar data extracted for each of them, which is critical for the review process (21). As in the previous stages, both authors engaged in document analysis, with one primarily responsible for inputting data into the extraction form, while the other oversaw and refined the information by reviewing the full texts of the selected studies.

Once the documents were coded into the data extraction form, a constant data comparison approach was implemented to identify the main patterns and lines of research by iteratively comparing the studies and collaborative discussing them between the two authors undertaking the task (21, 22). The results of this process are presented in the results section, where the literature review findings are grouped by topic.

## 3 Results: effectiveness discussion in the OSH literature

The literature review examined current research streams focusing on understanding successful interventions that can improve workplace OSH management. For these reasons, the selected documents address effectiveness from different perspectives by including both theoretical analyses of interventions' effectiveness and practical studies from real-world applications.

Through a comprehensive analysis of these documents, the review identified a highly debated topic embedded in the discussion of OSH interventions—the OSH Management Systems (OSHMSs)—to which a sub-section is dedicated. It is worth noting that in the OSH field, a prominent part of OSH interventions relies on OSHMSs, which are designed to foster improvement in OSH management at the organizational level. This connection emphasizes the significance of delving into OSHMSs when discussing OSH interventions, making them a natural area of investigation within the discussion of OSH interventions.

Before reviewing the literature, definitions of OSH interventions and OSHMSs are stated below.

- OSH interventions are actions taken to prevent injuries and diseases in the work environment by improving employees' safety, health, and wellbeing.
- OSHMSs do not share a consensus on what they are (14). The OSHMSs are either mandatory or voluntary (14, 23). Mandatory OSHMSs are developed from government legislation, and their use is enforced through inspections, fines, etc., as specified by the EU Directive 89/391/EEC (24). Voluntary OSHMSs are established to guide action at the national and enterprise levels, although they are not intended to replace national regulations. ILO (25) defined a voluntary OSHMS as: “A set of interrelated or interacting elements to establish OSH policy and objectives, and to achieve those objectives.” Frick et al. (26) defined a voluntary OSHMS as a comprehensive framework for policy development, risk assessment, risk management, and evaluation of effectiveness within an organization. In addition, every employer should establish a voluntary OSHMS in their workplace to better manage occupational accidents and diseases and continuously improve OSH performance (25). OSHMSs usually arise through private enterprises, employer groups, the government and its agencies, insurance carriers, professional organizations, and standards associations. The introduction of international standards, such as the ISO 45001:2018 (27), moves in this direction by providing frameworks for OSHMSs to manage risks and opportunities.

The following sections cover the effectiveness of OSH interventions (Section 3.1) and OSHMSs (Section 3.2), and Table 1 summarizes the essential findings.

**Table 1:** Findings on the effectiveness of OSH interventions and OSHMSs.

**OSH interventions**
Planning all the phases of interventions from the initial design to the ongoing monitoring for durable positive effects
Considering the complexity of the environment where interventions take place
Viewing context as a dynamic and essential part of the intervention process
Adopting a realist perspective, considering the mechanisms that positively or negatively affect interventions
Rising interest in methodologies, such as the program theory, which investigates the context and mechanisms influencing intervention development and outcomes
Continuous monitoring of intervention effects rather than time-limited evaluations
**OSHMSs**
Exploring OSHMSs for improving workplace OSH management and enhancing intervention outcomes
Enhancing OSHMSs in organizations with • Alignment with internal organizational culture and management • Management commitment and effective leadership • Workers' awareness and active involvement • Engagement with external entities, such as collaborative relationships with trade unions
Fostering collaboration between policymakers and OSH stakeholders for balanced perspectives on regulations
Promoting the benefits of self-regulation as a complementary approach to OSHMSs, by developing guidelines and frameworks that can facilitate the smooth integration of self-regulation within existing OSHMS structures
Encouraging organizations to adopt and regularly review evaluation criteria and Key Performance Indicators (KPIs) to assess OSHMS effectiveness and drive continuous improvement

### 3.1 Effectiveness of OSH interventions

A significant segment of the OSH literature focuses on the evaluation of OSH interventions to detect how they have (or should have) effectively contributed to improving OSH work conditions and a few examples are reported below. Micheli et al. (28) conducted research aiming to understand the mechanisms determining the success or failure of OSH interventions, considering both barriers and drivers along with contextual factors. Utilizing a multiple case study approach, 58 techno-organizational interventions were evaluated to assess the key factors influencing the interventions' outcomes. In another study, Olsen et al. (29) showed how the application of realist analysis and program theory to a specific New Zealand intervention could be generally used as a framework for evaluating, developing, and improving other national interventions. Fridrich et al. (15), as another example, introduced a Context, Process, and Outcome (CPO) evaluation model designed to assess complex organizational health interventions (OHIs), which was tested in an OHI at a Swiss hospital.

General interventions, potentially applicable to several working environments, have often been described in the literature (6, 14, 15, 28, 30–39). Other studies, attempting to develop more effective interventions, have targeted specific working contexts, such as Small and Medium-sized Enterprises (SMEs), which are normally more vulnerable than larger organizations and require *ad hoc* measures (6, 7, 40, 41). Other studies have examined precise typologies of interventions, such as musculoskeletal disorders (42) and training (43, 44).

Several scholars have expressed concerns about the limited guidance provided for building up effective interventions (13, 28, 36, 38). In this regard, several systematic literature reviews on OSH interventions have aimed to detect possible categories of interventions with higher effectiveness (7, 14, 30, 35, 44, 45). However, most of these reviews concluded that there were little to no quantitative results to assess the effectiveness of interventions. Owing to the considerable variability in the environment, interventions often exhibited a high degree of heterogeneity, making systematic comparisons challenging in literature reviews (14, 44, 45).

Predicting the true impact of interventions can be challenging (15) as their success is likely to depend on various factors such as their nature, specific workplace characteristics, and the broader external environment (14). Typically, these interventions are assessed under controlled ideal conditions, leading to outcomes that may not always meet expectations (28, 46). As such, further research is needed to enhance the effectiveness of these interventions, an aspect that sometimes does not receive the attention it deserves (35, 47–49). In this vein, scholars have questioned the appropriate methodology for evaluating interventions, and some have highlighted challenges linked to the diverse results (due to the high heterogeneity of the results) seen in quantitative evaluations. Some systematic literature reviews have implicitly emphasized the need to understand the mechanisms (what has or has not worked) that positively or negatively affect interventions (9, 14, 30, 33). Recent studies have been exploring alternative methods to evaluate and compare interventions, moving away from the commonly used Randomized Control Trials (RCTs). Instead, there is a growing interest in methodologies grounded in program theory (20). This allows the analysis of interventions through a more qualitative approach by considering the chains of events that affect their development and effectiveness. Thus, similar interventions can lead to divergent results because several contextual factors and mechanisms can affect the outcome, leading to success or failure. As Zwetsloot et al. (6) pointed out: “Whether OSH implementation will be successful depends on mechanisms, the characteristics of organizations, and their context.” In this vein, recent scholarly studies, such as Hale (50), Pryor et al. (51), and Uhrenholdt Madsen et al. (52), have focused on the roles of various OSH stakeholders. Zwetsloot et al. (6) and Hasle et al. (53) have explored the orchestration of these diverse stakeholders aiming to identify potential improvement areas beyond the confines of individual organizations. Key stakeholders, including representatives from trade unions and employer associations, play a pivotal role in shaping interventions that are well-grounded in real settings (54).

Furthermore, several scholars have proposed models based on program theory both for designing (16, 28, 55, 56) and evaluating (6, 15, 16, 28, 29, 39, 57) OSH interventions. Notably, Fridrich et al. (15) introduced a perspective that views the “context not only as a static and confounding factor that hinders or facilitates the implementation process but also as a transformable and essential part of the intervention.” Outcome evaluation is thus seen as a continuous process rather than a particular, time-limited intervention phase. This provides a dynamic view of program theory, enabling the monitoring of intervention effects over time, which is rarely performed. However, little evidence of the sustained positive impacts of such interventions over the long term has been provided in the literature (13).

Therefore, further research is required, and the OSHMSs presented below, from various angles, hold promise for enhancing workplace OSH management and potentially amplifying the positive effects of OSH interventions.

### 3.2 Effectiveness of OSHMSs

**Figure 2:**
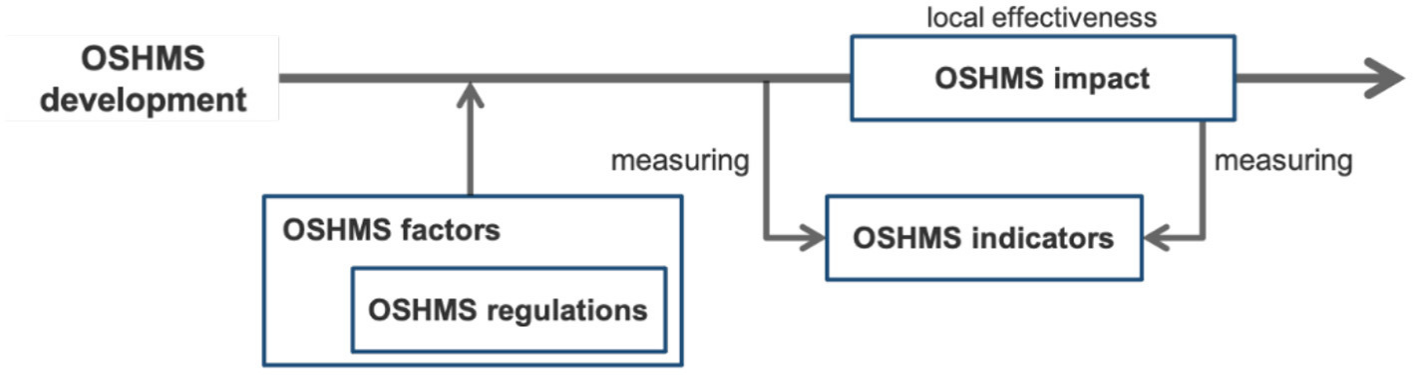
The relationship between macro-research areas on OSHMSs.

Through the review of OSHMS's literature, macro-research areas were identified (Figure 2), and the findings are presented following the classification below.

- OSHMS impact, i.e., the estimated impact on organizations.
- OSHMS factors are elements that can facilitate (drivers) or hinder (barriers) OSHMSs' development.
- OSHMS regulations, i.e., the role played by laws and regulations.
- OSHMS indicators, i.e., how the effectiveness of OSHMs should be assessed and measured.

Each of these categories highlights significant areas that previous scholars have identified and begun to address in recent years, contributing to a better understanding of OSHMSs and their effects on workplace OSH management. The results from the literature are presented below, highlighting both challenges and promising opportunities related to OSHMSs.

#### 3.2.1 OSHMS impact

Regarding OSHMS impact, a significant amount of literature referred to voluntary OSHMSs, especially international standards—i.e., OHSAS 18001:2007 (58), ISO 45001:2018 (27)—by assessing the estimated impact of those strategies on organizations. Two principal lines of thought exist regarding the impact of OSHMS standards.

- A positive effect, i.e., higher OSH performance (59–70).
- A neutral effect since a certification does not guarantee better OSH performance (71–80).

Scholars have highlighted the positive impacts of OSHMSs on organizations, particularly emphasizing two major aspects: OSH performance like work-related and fatal accident rates (63, 66, 67) and financial performance such as sales growth, enhanced labor productivity, and reduced accident-related expenses (59, 60, 68). While none stated that OSHMSs negatively affect an organization's OSH performance, it is recognized that simply obtaining a voluntary OSHMS certification does not necessarily imply better organizational OSH performance, since it needs to be sustained by the organization's culture and management (71). Furthermore, OSHMSs might sometimes address generic concerns rather than the specific needs of an organization (72, 80).

OSHMS audits are generally well-perceived and have the potential to be transformative tools, but, in some cases, become “a ritual rather than a means of improving workplace health and safety” (73). Notably, certified OSHMS adopters generally provide a higher level of OSH management than non-adopters. However, there are instances where the actual efforts toward OSH within certified organizations may seem less pronounced, suggesting that certification alone does not ensure a high level of OSH management for all adopters (70). Yet, the direct correlation between such certifications and enhanced OSH performance is not always linear (26). Building on this, Frick (81) outlined three integral components that define a robust OSHMS: procedures for risk assessment (what should be done), empowering stakeholders to implement procedures (how to do it), and management control (doing the right thing).

#### 3.2.2 OSHMS factors

The effective implementation of OSHMSs relies on several factors that can facilitate or hinder their development. A comprehensive review by da Silva and Amaral (82) has provided a consistent number of OSHMS factors, those contributing to the success of OSHMSs and other potential obstacles in their implementation. This analysis was further enriched by incorporating insights from other scholarly studies to integrate and confirm the initially identified factors. Table 2 offers a consolidated overview, summarizing all the drivers and barriers associated with OSHMS implementation.

**Table 2:** Drivers and barriers to OSHMS implementation.

**OSHMS drivers**		**OSHMS barriers**	
**Organization commitment**		**Organization commitment**	
Workers' awareness	(83)	Lack of management commitment	(82, 84, 85)
Workers' participation	(82, 84–87)	Lack of knowledge regarding the importance of OSHMSs, particularly in SMEs	(82, 88, 89)
Management commitment and leadership	(66, 82, 84–87, 90, 91)	Differing visions between organization managers and OSH managers	(63, 82)
		Lack of workers' participation	(82, 84)
		Lack of safety culture	(82, 84)
**Organization synergies**		**Availability of resources**	
Trade unions' involvement	(85)	Lack of specialized personnel	(82, 88, 92)
More participatory and flatter organizational structures	(93)	Lack of economic resources, particularly in SMEs	(82, 88, 89)
Cooperative relations among labor-market	(93)	Lack of time (time-wasting), particularly in SMEs	(89)
**OSH managemen**t		**OSH management**	
Training	(82, 84, 86, 93)	Often underestimate the risks within organizations, particularly in SMEs	(89)
Risk assessment	(82, 86)	Lack of safe communication	(82, 84)
Definition of responsibilities	(82, 86)		
Communication and dissemination of results	(82, 86)		
OSH policy and programs	(82, 84, 91, 94)		
Supervision	(82, 90)		
Safe work procedures	(82, 90)		

The commitment of an organization and its approach to OSH management play a crucial role in facilitating or hindering OSHMS' development. Management commitment and good leadership (66, 82, 84–87, 90, 91) on one side and workers' awareness (83) and active participation (82, 84–87) on the other are core drivers encouraging the establishment of OSHMSs. Positive OSH management strategies, such as transparent communication, robust risk assessments, and proactive supervision, act as drivers, while their neglect or mismanagement can be deterrents (82). Furthermore, the alignment of OSHMS efforts with other internal—like fostering a more inclusive organizational structure (93)—and external—like collaborating with trade unions (85)—processes is always welcome, and organizations can leverage them. Concerning external factors, Rocha (93) brought a fresh perspective by examining how national institutional settings influence OSHMS dynamics and recognizing that beyond an organization's internal capacities, the broader national context also plays a crucial role in shaping OSHMS outcomes. Consequently, organizations in different countries should deal differently with OSHMS requirements.

#### 3.2.3 OSHMS regulations

Another stream of the literature focuses on the interplay of OSHMSs' effectiveness and regulations. While laws and regulations can sometimes be perceived as obstacles to the development of OSHMSs, their true value on OSH performance can be perceived when effectively managed and implemented (95). For instance, Hale and Swuste (96) called regulations “invisible barriers,” and Hollnagel (97) considered legislation as an “invisible barrier system.” There is a tendency for policymakers to have optimistic views about how mandatory OSHMSs operate (95). Hale et al. (98) viewed self-regulation—the application of voluntary norms and codes of good practice—as a way to reduce the perceived regulatory pressures on OSHMSs.

#### 3.2.4 OSHMS indicators

To foster confidence in OSHMSs and motivate organizations to adopt them, it is essential to establish clear evaluation criteria and Key Performance Indicators (KPIs) (64, 99). Three studies have been selected that identified optimal KPIs for OSHMSs (64, 82, 100). Podgórski (100), for instance, applied the Analytic Hierarchy Process (AHP) to select 20 KPIs out of a larger set of candidate indicators (109), categorized under areas such as Policy, Organizing, Planning and Implementation, Evaluation, and Action for Improvement.

## 4 Discussion: status quo and implications

The results of the literature review indicated that scholars wondered about the effectiveness of interventions, including OSHMSs, developed for OSH improvement.

### 4.1 About OSH interventions

Considering OSH interventions, practitioners have begun to describe several field interventions from an operational perspective. However, drawing broad conclusions from the literature has seldom been possible due to the unique dynamics at play. Several systematic literature reviews have analyzed interventions in an attempt to identify common threads and strategies to achieve higher effectiveness, but the diverse and varied environments often rendered them too distinct for direct comparison. Different theoretical lenses among researchers may indeed prevent the comparison of similar interventions. A critical realism perspective offers a promising approach to examining OSH interventions. The realist analysis, consistent with the above epistemological perspective, is rooted in understanding the underlying causal chains of events and their effects—essentially, discerning what works, for whom, under what conditions, and how (20). This aligns with the literature, where scholars have underlined the value of studying the mechanisms—what has or has not worked—of the interventions (14, 30, 33).

Given the above considerations, it is worth analyzing interventions not as black boxes but considering the different factors affecting them (28). Contextual factors play a paramount role in all phases of the design, implementation, and evaluation of interventions (15). Giving attention to these factors can enhance the probability of achieving desired outcomes. Quantitative assessments may not always be the most suitable or feasible for OSH interventions, as quantifiable data are rarely tracked and often difficult to retrieve. It is important to understand that qualitative methods can be equally insightful and, in certain settings, may be more appropriate.

The design, implementation, and evaluation phases should be equally considered, while processes with greater short-term benefits are still often prioritized, inhibiting the crucial final evaluation phase. Indeed, interventions should be evaluated in advance, and the study of *ad hoc* indicators would enable long-term monitoring of the impact of interventions (13). In addition, monitoring induces the development of more effective interventions that rely on grounded knowledge (29). Therefore, it is crucial to ensure continuity between interventions to gain mutual benefits and contribute value at the system level. Moreover, from a perspective of fostering human-centered technological development, there is a pressing need to transition from interventions that prioritize economic maximization to interventions that seek to reconcile the human, social, and environmental implications with economic-financial considerations.

The newly released ISO 45001:2018 (27) takes this direction by moving from a focus on individual system components toward a better understanding of the entire socio-technical system—i.e., multidirectional interactions and information flow across the system, networks of stakeholders and their interdependency, and the effects of internal and external factors and constraints (101).

### 4.2 About OSHMS impact

The second macro-area of the literature review included the implementation of OSHMSs in practice for OSH improvement, and similar considerations to OSH interventions applied to their effectiveness. Their development is comparable to that of field interventions and is generally more structured, long-lasting, and potentially more powerful when implemented in organizations. In Section 3.2, four macro-areas of research were detected for OSHMSs—their impact, influential factors, regulatory aspects, and performance indicators—and insights for effective OSHMSs were gathered.

Effective OSHMSs should ensure safe and healthy workplaces by continuously improving the OSH performance of organizations (25). The literature analysis highlighted that there is considerable research on OSH to study OSHMSs, their relationship with the surrounding environment, and the affecting factors. Although several studies explored ways to enhance the performance of OSHMSs and their potential is recognized, there remains a gap in understanding their tangible effectiveness at the organizational level. This presents an opportunity for both scholars and practitioners to delve deeper into this area of research.

### 4.3 About OSHMS regulations

The literature suggests that having certification is valuable, but it alone does not guarantee optimal effectiveness within a specific context. Certification is an important tool for organizations to ensure better performance; however, positive organizational culture and management are required (71).

“Regulatory burden” is a common periphrasis in the literature, which is clear proof that regulations are often perceived as potential barriers or “invisible barriers” (96) to OSH improvement. However, collaboration between organizations and policymakers can pave the way for more tailored and effective solutions. As national authorities increasingly recognize the importance of this collaboration, it presents an opportunity for both policymakers and organizations. Policymakers can secure interventions to prevent them from becoming backburners, and organizations can be incentivized to join such interventions by developing something that would fit well with their specificities.

### 4.4 About OSHMS factors

Other studies on OSHMSs have highlighted the key factors that promote their development and those that might pose challenges. As outlined in Table 2, there are internal factors related to the organization's structure and external factors influenced by the outer environment. Some characteristics have been identified both as potential barriers and drivers. Identified factors are often classified by their characteristics as barriers or drivers and by considering whether their presence or absence facilitates or inhibits the development of OSHMSs. For example, a strong management commitment is vital for successful OSHMS implementation, while its unavailability is considered a barrier. Based on Kano et al.'s theory (102), these factors can be divided into three major categories: must-haves, performers, and delighters. Factors simply evaluated as barriers can be considered must-have requirements that would hinder the development of OSHMSs or interventions in general, such as a lack of time and resources (82, 88, 89). Most of these factors are regarded as performers because their existence can change the actual deployment of OSHMSs. For example, good leadership can support collaboration between individuals and, therefore, the OSHMSs' work, whereas bad leadership can inhibit their implementation. Lastly, delighters represent the factors that represent true value added. As highlighted by Frick (85), the involvement of trade unions offers invaluable insights and is a key driver for employers to leverage their field knowledge to build new competencies at the organizational level. Key stakeholders, including representatives from trade unions and employer associations, play a pivotal role in shaping interventions that are well-grounded in real settings.

### 4.5 About OSHMS indicators

In a similar vein, a few studies have looked at KPIs to measure the effectiveness of OSHMSs. As for OSH interventions, *ad hoc* indicators offer a means for longitudinally assessing OSHMS impact, enabling continuous monitoring of OSHMS effectiveness and also promoting the development of more robust systems. Indeed, by leveraging insights from prior OSHMS implementations, organizations can refine their practices, adhere to industry best standards, and mitigate the risk of unsuccessful initiatives.

### 4.6 Implications

As shown in the literature, higher coordination between OSH stakeholders improves the effectiveness of OSHMSs by increasing their adoption levels and, in general, OSH interventions (54, 93). The EU-OSHA's ESENER report (103) emphasizes this element, noting that the “presence (and involvement) of employee representation is a factor in ensuring that such OSH policies and action plans are put into practice.” A conscious network of stakeholders is indeed vital to OSH improvement, which would enable any implemented intervention to be sustainable and effective in the long run (54). In addition, the broader environment, encompassing various contextual factors, plays a pivotal role in shaping national OSH management. National laws determine the key differences between nations concerning stakeholder involvement, functions, and more. As noted by Rocha (93), institutional effects strongly affect the OSH network of stakeholders and often remain relatively stable in the short term. Consequently, organizations across different nations should deal with these requirements differently, thus requiring tailored approaches.

Table 3 summarizes the above discussion by revealing the status quo of the effectiveness of OSH interventions and suggesting future research directions.

**Table 3:** Status quo of OSH interventions' effectiveness and research directions.

**Status quo**	**Research directions**
**OSH interventions**	
There is increasing attention to all the phases of interventions' development—design, implementation, and evaluation—but their effectiveness requires sufficient proof.	Higher attention should be paid to the evaluation of OSH interventions, which is still less developed than the other two phases of design and implementation. The effectiveness of interventions should be measured through clear indicators before and soon after their end and their impact should be monitored over time. Indeed, the study of *ad-hoc* indicators would enable long-term monitoring of interventions' impact and their monitoring induces the development of future, more effective, interventions that rely on grounded knowledge.
**OSHMS impact**	
OSHMSs have been believed to ensure safe and healthy workplaces by continuously improving the OSH performance of organizations. There is considerable research studying OSHMSs, their relations with the surrounding environment, and the affecting factors, but low evidence of their tangible effectiveness at the organizational level.	Further research on the determinants—factors, indicators, regulations—that make OSHMSs more impactful at the organizational level is required.
**OSHMS regulations**	
Regulations are predominantly viewed as barriers to OSH improvement. Organizations view regulations as top-down directives not properly working in their environment. Literature has been questioning ways for improvement.	Being familiar with regulations makes OSHMSs more effective in the environment, thus leveraging enabling factors and controlling hindering factors Working with policymakers could be an effective strategy for the medium and long term to entice organizations to work for shared solutions, causing regulations no longer to be viewed as top-down directives. Collaboration between OSH stakeholders at various levels is the key. National authorities are moving in that direction; thus, developing interventions for OSH improvement that involve stakeholders in the field.
**OSHMS factors**	
There are enabling factors (drivers) and potential barriers that can inhibit the development of OSHMSs. Internal factors, related to the organization's structure, and external factors, depending on the outer environment, are being studied. For example, a positive organization's culture and management can foster better performance in OSHMSs.	The development of OSHMSs should consider all inherent dynamics of the environment in which they are implemented. Enabling factors (drivers) and potential barriers of OSHMSs have been identified; however, how to foster drivers and inhibit barriers in real OSHMSs has not been studied. It is unclear whether such factors generate synergies and trade-offs when combined.
**OSHMS indicators**	
The best set of KPIs has been enquired from researchers (e.g., by applying the AHP to select KPIs out of a larger set of candidate indicators).	The same considerations for OSHMS factors apply to indicators. Scholars have started to identify sets of KPIs for OSHMS assessment and monitoring; however, they remain untested in the real field. Indicators enabling the monitoring over time of implemented OSHMSs should be encouraged.

## 5 Limitations

This study has a few limitations that stem from the integrative process of the literature review, which, beyond the benefits detailed in Section 2, entails some inherent limitations. Although reliability has been secured by providing details on the entire process of the literature review, from the search to the analysis and categorization of data, the replicability of the results is not as strong as for systematic literature reviews, where the process is fully falsifiable. The use of search protocols increases the replicability of the process by providing an initial batch of documents to be evaluated and identifying initial literature clusters.

The Authors believe that the applied literature review process enabled the results to reach a satisfactory level of comprehensiveness and exhaustiveness, which was otherwise unattainable through a systematic approach. The integrative literature review does not claim to be exhaustive in terms of the included documents, as it might have potentially turned into an endless process, but rather, consistently pursues the objective of the research by combining different perspectives, obtaining relevant results, and keeping the number of documents affordable (17, 18).

In support of this, several studies in the OSH literature, such as Dyreborg et al. (9), Fridrich et al. (15), and Hasle et al. (55), have shown that systematic literature reviews may be unsuccessful in finding robust results due to high heterogeneity and lack of available standardized data. Research on health and safety has great potential because human-based science focuses on several thematic areas that address OSH issues from a multitude of aspects. However, this increases the amount of potentially retrievable information and the number of pertinent documents. Another direct consequence is the low awareness of keywords, which often have several synonyms, and their meanings might differ. For example, construction activities implemented in the workplace are predominantly called “projects,” whereas in the manufacturing industry, “intervention” is the most common term. In addition, because the literature has shown low topic categorization, there are no available frameworks for data classification. Therefore, this study suggested a straightforward structure to read the results by defining a fil rouge between OSH interventions and OSHMSs and their evaluation of effectiveness, which was constructed by iteratively comparing documents according to a data comparison approach (22).

Similar to most exploratory studies, the results cannot be considered exhaustive; instead, they enable the identification of patterns that might be beneficial to future research because they are still underdeveloped and have high potential. Consequently, other literature analyses are suggested to further explore and validate the findings of this study.

## 6 Conclusions

Through an integrative review of OSH literature, this study examined extant research contributions to the effectiveness of OSH interventions, including OSHMSs, by revealing their status quo and identifying room for improvement.

Studying appropriate ways to develop interventions is currently a matter of discussion. Since its inception, many steps have been taken; however, there is still a long road ahead. The literature includes several analyses of interventions implemented in the workplace and specifically, the OSHMSs for OSH improvement. Their implementation in organizations might be comparable to that of field OSH interventions, and they are generally more structured, long-lasting, and potentially more powerful. Their potential is high, as shown by theoretical research, yet there remains a rich vein of exploration regarding their real-world deployment within workplaces.

Based on the literature, some research streams for future developments in OSH interventions were identified and summarized in Table 3. In particular, coordination among OSH stakeholders, knowledge awareness, and information sharing are only a few drivers that can improve the effectiveness of field OSH interventions. A conscious network of stakeholders would support the development of interventions and work toward continuous improvement (54). A recent EU-OSHA report (104) shares the same view by stating that orchestrated/coordinated actions may be more effective than unilateral ones in leveraging better OSH, but strong leadership is required. Further research in this direction is highly recommended and, except for a few studies, such as Hasle et al. (53) and Zwetsloot et al. (6), it is an unexplored path with high potential.

The Authors hope that future research will pursue the proposed directions that, from different perspectives, would support OSH improvement through interventions that leverage more structured processes and encourage organizations to be proactive in daily OSH management.

## Author contributions

GV: Conceptualization, Data curation, Formal analysis, Investigation, Methodology, Supervision, Validation, Writing – original draft, Writing – review & editing, Project administration. GM: Conceptualization, Methodology, Validation, Writing – original draft, Writing – review & editing, Investigation, Project administration.

## References

[1] ILO. International Labour Organization (2023). Available online at: https://www.ilo.org/global/lang–en/index.htm (accessed December 22, 2023).

[2] ILO. Safety and health at work (2023). Available online at: https://www.ilo.org/global/topics/safety-and-health-at-work/lang–de/index.htm (accessed December 22, 2023).

[3] Lloyd's Register Foundation. Mapping Risk A Review of Global Data Sources on Safety and Risk. London, United Kingdom. (2019). Available online at: www.lrfoundation.org.uk (accessed December 22, 2023).

[4] ILO. World Statistic - The enormous burden of poor working conditions. (2023). Available online at: http://www.ilo.org/moscow/areas-of-work/occupational-safety-and-health/WCMS_249278/lang–en/index.htm (accessed December 22, 2023).

[5] Punnett L Cavallari JM Henning RA Nobrega S Dugan AG Cherniack MG. Defining “integration” for total Worker Health^®^: a new proposal. Ann Work Expo Health. (2020) 64:223–35. 10.1093/annweh/wxaa003 32003780 PMC7064271

[6] Zwetsloot GIJM Schmitt-Howe B Nielsen KT. Success factors for OSH implementation. Opening the black box of OSH realization. Policy Pract Health Saf. (2020) 18:196–210. 10.1080/14773996.2020.1786994

[7] Curtis Breslin F Kyle N Bigelow P Irvin E Morassaei S MacEachen E. Effectiveness of health and safety in small enterprises: a systematic review of quantitative evaluations of interventions. J Occup Rehabil. (2010) 20:163–79. 10.1007/s10926-009-9212-1 19908131

[8] ILO. Technical and Ethical Guidelines for Workers' Health Surveillance (Occupational Safety and Health Series No. 72). Geneva (1998).

[9] Dyreborg J Lipscomb HJ Nielsen K Törner M Rasmussen K Frydendall KB. Safety interventions for the prevention of accidents at work: a systematic review. Campbell Syst Rev. (2022) 18:1–187. 10.1002/cl2.1234 36911341 PMC9159701

[10] Rodrigues MA Sá A Masi D Oliveira A Boustras G Leka S. Occupational health & safety (OHS) management practices in micro- and small-sized enterprises: the case of the Portuguese waste management sector. Saf Sci. (2020) 129:104794. 10.1016/j.ssci.2020.104794

[11] Masi D Cagno E. Barriers to OHS interventions in small and medium-sized enterprises. Saf Sci. (2015) 71:226–41. 10.1016/j.ssci.2014.05.020 26654679

[12] Micheli GJL Farné S Vitrano G. A holistic view and evaluation of health and safety at work: enabling the assessment of the overall burden. Saf Sci. (2022) 156:105900. 10.1016/j.ssci.2022.105900

[13] Vitrano G Micheli GJL Guglielmi A De Merich D Pellicci M Urso D. Sustainable occupational safety and health interventions: a study on the factors for an effective design. Saf Sci. (2023) 166:106249. 10.1016/j.ssci.2023.106249

[14] Robson LS Clarke JA Cullen K Bielecky A Severin C Bigelow PL. The effectiveness of occupational health and safety management system interventions: a systematic review. Saf Sci. (2007) 45:329–53. 10.1016/j.ssci.2006.07.003

[15] Fridrich A Jenny GJ Bauer GF. The context, process, and outcome evaluation model for organisational health interventions. Biomed Res Int. (2015) 2015:414832. 10.1155/2015/414832 26557665 PMC4628757

[16] von Thiele Schwarz U Nielsen K Edwards K Hasson H Ipsen C Savage C. How to design, implement and evaluate organizational interventions for maximum impact: the Sigtuna Principles. Eur J Work Organ Psychol. (2021) 30:415–27. 10.1080/1359432X.2020.1803960 34518756 PMC8432268

[17] Snyder H. Literature review as a research methodology: an overview and guidelines. J Bus Res. (2019) 104:333–9. 10.1016/j.jbusres.2019.07.039

[18] Torraco RJ. Writing integrative literature reviews: guidelines and examples. Hum Resour Dev Rev. (2005) 4:356–67. 10.1177/1534484305278283

[19] Walters D Wadsworth E Hasle P Refslund B Ramioul M. Safety and health in micro and small enterprises in the EU: final report from the 3-year SESAME project European Agency for Safety and Health at Work. Luxembourg (2018).

[20] Pawson R. Evidence-Based Policy: A Realist Perspective. London: SAGE Publications (2006). 10.4135/9781849209120

[21] Whittemore R Knafl K. The integrative review: updated methodology. J Adv Nurs. (2005) 52:546–53. 10.1111/j.1365-2648.2005.03621.x 16268861

[22] Glaser BG. The constant comparative method of qualitative analysis. Soc Probl. (1965) 12:436–45. 10.1525/sp.1965.12.4.03a00070

[23] Frick K Kempa V. Occupational health & safety management system - when are they good for your health? Brussels: European Trade Union Institute (ETUI) (2011).

[24] European Council. European Directive 89/391/EEC of 12 June: introduction of measures to encourage improvements in the safety and health of workers at work (1989). Available online at: https://eur-lex.europa.eu/eli/dir/1989/391/oj

[25] ILO-OSH. Guidelines on occupational safety and health management systems. Geneva. (2001). Available online at: https://www.ilo.org/safework/info/standards-and-instruments/WCMS_107727/lang–en/index.html (accessed December 22, 2023).

[26] Frick K Jensen PL Quinlan M Wilthagen T. Systematic Occupational Health and Safety Management - Perspectives on an International Development. Oxford: Pergamon (2000).

[27] ISO. Occupational health and safety management systems — Requirements with guidance for use (ISO 45001:2018). The International Organization for Standardization (ISO) (2018).38131694

[28] Micheli GJL Cagno E Calabrese A. The transition from occupational safety and health (OSH) interventions to OSH outcomes: an empirical analysis of mechanisms and contextual factors within small and medium-sized enterprises. Int J Environ Res Public Health. (2018) 15:1621. 10.3390/ijerph15081621 30065234 PMC6122035

[29] Olsen K Legg S Hasle P. How to use programme theory to evaluate the effectiveness of schemes designed to improve the work environment in small businesses. Work. (2012) 41:5999–6006. 10.3233/WOR-2012-0036-5999 22317740

[30] Cooklin A Joss N Husser E Oldenburg B. Integrated approaches to occupational health and safety: a systematic review. Am J Health Promot. (2017) 31:401–12. 10.4278/ajhp.141027-LIT-542 26730561

[31] Whysall Z Haslam C Haslam R. Implementing health and safety interventions in the workplace: an exploratory study. Int J Ind Ergon. (2006) 36:809–18. 10.1016/j.ergon.2006.06.007

[32] Whysall Z Haslam C Haslam R. A stage of change approach to reducing occupational ill health. Prev Med. (2006) 43:422–8. 10.1016/j.ypmed.2006.07.004 16908057

[33] Goldenhar LM Schulte PA. Methodological issues for intervention research in occupational health and safety. Am J Ind Med. (1996) 29:289–94. 10.1002/(SICI)1097-0274(199604)29:4&lt;289::AID-AJIM2&gt;3.0.CO;2-K 8728126

[34] Lee G. A systematic review of occupational health and safety business cases. Workplace Health Saf. (2018) 66:95–104. 10.1177/2165079917730073 29117804

[35] Teufer B Ebenberger A Affengruber L Kien C Klerings I Szelag M. Evidence-based occupational health and safety interventions: a comprehensive overview of reviews. BMJ Open. (2019) 9:e032528. 10.1136/bmjopen-2019-032528 31831544 PMC6924871

[36] Andersen JH Malmros P Ebbehoej NE Flachs EM Bengtsen E Bonde JP. Systematic literature review on the effects of occupational safety and health (OSH) interventions at the workplace. Scand J Work Environ Health. (2019) 45:103–13. 10.5271/sjweh.3775 30370910

[37] Fan D Lo CKY Ching V Kan CW. Occupational health and safety issues in operations management: a systematic and citation network analysis review. Int J Prod Econ. (2014) 158:334–44. 10.1016/j.ijpe.2014.07.025

[38] Hale AR Guldenmund FW van Loenhout PLCH Oh JIH. Evaluating safety management and culture interventions to improve safety: effective intervention strategies. Saf Sci. (2010) 48:1026–35. 10.1016/j.ssci.2009.05.006

[39] Pedersen LM Nielsen KJ Kines P. Realistic evaluation as a new way to design and evaluate occupational safety interventions. Saf Sci. (2012) 50:48–54. 10.1016/j.ssci.2011.06.010

[40] Masi D Cagno E Micheli GJL. Developing, implementing and evaluating OSH interventions in SMEs: a pilot, exploratory study. Int J Occup Saf Ergon. (2014) 20:385–405. 10.1080/10803548.2014.11077059 25189744

[41] Cagno E Micheli GJL Jacinto C Masi D. An interpretive model of occupational safety performance for small- and medium-sized enterprises. Int J Ind Ergon. (2014) 44:60–74. 10.1016/j.ergon.2013.08.005

[42] Yazdani A Wells R. Barriers for implementation of successful change to prevent musculoskeletal disorders and how to systematically address them. Appl Ergon. (2018) 73:122–40. 10.1016/j.apergo.2018.05.004 30098627

[43] Ricci F Chiesi A Bisio C Panari C Pelosi A. Effectiveness of occupational health and safety training: a systematic review with meta-analysis. J Workplace Learn. (2016) 28:355–77. 10.1108/JWL-11-2015-0087

[44] Robson LS Stephenson CM Schulte PA Amick BC Irvin EL Eggerth DE. A systematic review of the effectiveness of occupational health and safety training. Scand J Work Environ Health. (2012) 38:193–208. 10.5271/sjweh.3259 22045515

[45] Verbeek J Ivanov I. Essential occupational safety and health interventions for low- and middle-income countries: an overview of the evidence. Saf Health Work. (2013) 4:77–83. 10.1016/j.shaw.2013.04.004 23961329 PMC3732143

[46] Kennedy CA Amick BC Dennerlein JT Brewer S Catli S Williams R. Systematic review of the role of occupational health and safety interventions in the prevention of upper extremity musculoskeletal symptoms, signs, disorders, injuries, claims and lost time. J Occup Rehabil. (2010) 20:127–62. 10.1007/s10926-009-9211-2 19885644

[47] Verbeek J. Could we have better occupational health guidelines, please? Scand J Work Environ Health. (2018) 44:441–2. 10.5271/sjweh.3764 30079429

[48] Baril-Gingras G Bellemare M Brun JP. The contribution of qualitative analyses of occupational health and safety interventions: an example through a study of external advisory interventions. Saf Sci. (2006) 44:851–74. 10.1016/j.ssci.2006.05.003

[49] Noy YI Hettinger LJ Dainoff MJ Carayon P Leveson NG Robertson MM. Editorial: emerging issues in sociotechnical systems thinking and workplace safety. Ergonomics. (2015) 58:543–7. 10.1080/00140139.2014.1001445 25819595 PMC4647650

[50] Hale A. From national to European frameworks for understanding the role of occupational health and safety (OHS) specialists. Saf Sci. (2019) 115:435–45. 10.1016/j.ssci.2019.01.011

[51] Pryor P Hale A Hudson D. Development of a global framework for OHS professional practice. Saf Sci. (2019) 117:404–16. 10.1016/j.ssci.2019.04.033

[52] Uhrenholdt Madsen C Hasle P Limborg HJ. Professionals without a profession: occupational safety and health professionals in Denmark. Saf Sci. (2019) 113:356–61. 10.1016/j.ssci.2018.12.010

[53] Hasle P Limborg HJ Grøn S Refslund B. Orchestration in work environment policy programs. Nord J Work Life Stud. (2017) 7:43–62. 10.18291/njwls.v7i3.97092

[54] De Merich D Gnoni MG Guglielmi A Micheli GJ Sala G Tornese F. Designing national systems to support the analysis and prevention of occupational fatal injuries: evidence from Italy. Saf Sci. (2022) 147:105615. 10.1016/j.ssci.2021.105615

[55] Hasle P Limborg HJ Nielsen KT. Working environment interventions - bridging the gap between policy instruments and practice. Saf Sci. (2014) 68:73–80. 10.1016/j.ssci.2014.02.014

[56] Hasle P Kvorning LV Rasmussen CDN Smith LH Flyvholm MA. A model for design of tailored working environment intervention programmes for small enterprises. Saf Health Work. (2012) 3:181–91. 10.5491/SHAW.2012.3.3.181 23019530 PMC3443693

[57] Vitrano G Micheli GJL Sala G Guglielmi A de Merich D Campo G. Programme theory evaluation of initiatives to support Health and Safety improvement: an Italian cross-sectional study. In: Proceedings of the XXIII Summer School “Francesco Turco” – Industrial Systems Engineering. Bergamo (2021).

[58] BSI. Occupational Health and Safety Management Systems. Requirements (BS OHSAS 18001:2007). London (2007).

[59] Abad J Lafuente E Vilajosana J. An assessment of the OHSAS 18001 certification process: objective drivers and consequences on safety performance and labour productivity. Saf Sci. (2013) 60:47–56. 10.1016/j.ssci.2013.06.011

[60] Lo CKY Pagell M Fan D Wiengarten F Yeung ACL. OHSAS 18001 certification and operating performance: the role of complexity and coupling. J Oper Manag. (2014) 32:268–80. 10.1016/j.jom.2014.04.004

[61] Bottani E Monica L Vignali G. Safety management systems: performance differences between adopters and non-adopters. Saf Sci. (2009) 47:155–62. 10.1016/j.ssci.2008.05.001

[62] Santos G Barros S Mendes F Lopes N. The main benefits associated with health and safety management systems certification in Portuguese small and medium enterprises post quality management system certification. Saf Sci. (2013) 51:29–36. 10.1016/j.ssci.2012.06.014

[63] Yoon SJ Lin HK Chen G Yi S Choi J Rui Z. Effect of occupational health and safety management system on work-related accident rate and differences of occupational health and safety management system awareness between managers in South Korea's construction industry. Saf Health Work. (2013) 4:201–9. 10.1016/j.shaw.2013.10.002 24422176 PMC3889079

[64] Mohammadfam I Kamalinia M Momeni M Golmohammadi R Hamidi Y Soltanian A. Evaluation of the quality of occupational health and safety management systems based on key performance indicators in certified organizations. Saf Health Work. (2017) 8:156–61. 10.1016/j.shaw.2016.09.001 28593071 PMC5447402

[65] Fan D Lo CKY. A tough pill to swallow?: the impact of voluntary occupational health and safety management system on firms' financial performance in fashion and textiles industries. J Fash Mark Manag Int J. (2012) 16:128–40. 10.1108/13612021211222798

[66] Autenrieth DA Brazile WJ Sandfort DR Douphrate DI Román-Muñiz IN Reynolds SJ. The associations between occupational health and safety management system programming level and prior injury and illness rates in the US dairy industry. Saf Sci. (2016) 84:108–16. 10.1016/j.ssci.2015.12.008 36407878 PMC9670018

[67] Bayram M Üngan MC. The relationships between OHS prevention costs, OHSMS practices, employee satisfaction, OHS performance and accident costs. Total Qual Manag Bus Excell. (2020) 31:1325–44. 10.1080/14783363.2018.148089727667202

[68] Yang M Maresova P. Adopting occupational health and safety management standards: the impact on financial performance in pharmaceutical firms in China. Risk Manag Healthc Policy. (2020) 13:1477–87. 10.2147/RMHP.S261136 32982506 PMC7490044

[69] Kim KW. Effect of an occupational health and safety management system based on KOSHA 18001 on industrial accidents. Work. (2021) 68:449–60. 10.3233/WOR-203385 33522993

[70] Uhrenholdt Madsen C Vester Thorsen S Hasle P Leonhardt Laursen L Dyreborg J. Differences in occupational health and safety efforts between adopters and non-adopters of certified occupational health and safety management systems. Saf Sci. (2022) 152:105794. 10.1016/j.ssci.2022.105794

[71] Granerud RL Rocha RS. Organisational learning and continuous improvement of health and safety in certified manufacturers. Saf Sci. (2011) 49:1030–9. 10.1016/j.ssci.2011.01.009

[72] Hohnen P Hasle P. Making work environment auditable - a “critical case” study of certified occupational health and safety management systems in Denmark. Saf Sci. (2011) 49:1022–9. 10.1016/j.ssci.2010.12.005

[73] Blewett V O'Keeffe V. Weighing the pig never made it heavier: auditing OHS, social auditing as verification of process in Australia. Saf Sci. (2011) 49:1014–21. 10.1016/j.ssci.2010.12.010

[74] Yazdani A Neumann WP Imbeau D Bigelow P Pagell M Theberge N. How compatible are participatory ergonomics programs with occupational health and safety management systems? Scand J Work Environ Health. (2015) 41:111–23. 10.5271/sjweh.3467 25380301

[75] Ghahramani A Salminen S. Evaluating effectiveness of OHSAS 18001 on safety performance in manufacturing companies in Iran. Saf Sci. (2019) 112:206–12. 10.1016/j.ssci.2018.10.021

[76] Ghahramani A. An investigation of safety climate in OHSAS 18001-certified and non-certified organizations. Int J Occup Saf Ergon. (2016) 22:414–21. 10.1080/10803548.2016.1155803 27108658

[77] Ghahramani A Summala H. A study of the effect of OHSAS 18001 on the occupational injury rate in Iran. Int J Inj Contr Saf Promot. (2017) 24:78–83. 10.1080/17457300.2015.1088038 26401723

[78] Simukonda W Manu P Mahamadu AM Dziekonski K. Occupational safety and health management in developing countries: a study of construction companies in Malawi. Int J Occup Saf Ergon. (2020) 26:303–18. 10.1080/10803548.2018.1482649 29846152

[79] Uhrenholdt Madsen C Kirkegaard ML Dyreborg J Hasle P. Making occupational health and safety management systems ‘work': a realist review of the OHSAS 18001 standard. Saf Sci. (2020) 129:104843. 10.1016/j.ssci.2020.104843

[80] Pinto C Colim A Domingues P Sampaio P Arezes P. Development of guidelines for an occupational health and safety management systems towards industry 40. Stud Syst Decis Control. (2023) 449:17–29. 10.1007/978-3-031-12547-8_2

[81] Frick K. The 50/50 implementation of Sweden's mandatory systematic work environment management. Policy Pract Health Saf. (2014) 12:23–46. 10.1080/14774003.2014.11667802

[82] da Silva SLC Amaral FG. Critical factors of success and barriers to the implementation of occupational health and safety management systems: a systematic review of literature. Saf Sci. (2019) 117:123–32. 10.1016/j.ssci.2019.03.026

[83] Ismail Z Doostdar S Harun Z. Factors influencing the implementation of a safety management system for construction sites. Saf Sci. (2012) 50:418–23. 10.1016/j.ssci.2011.10.001

[84] Ghahramani A. Factors that influence the maintenance and improvement of OHSAS 18001 in adopting companies: a qualitative study. J Clean Prod. (2016) 137:283–90. 10.1016/j.jclepro.2016.07.087

[85] Frick K. Worker influence on voluntary OHS management systems - a review of its ends and means. Saf Sci. (2011) 49:974–87. 10.1016/j.ssci.2011.04.007

[86] Mohammadfam I Kamalinia M Momeni M Golmohammadi R Hamidi Y Soltanian A. Developing an integrated decision making approach to assess and promote the effectiveness of occupational health and safety management systems. J Clean Prod. (2016) 127:119–33. 10.1016/j.jclepro.2016.03.123

[87] Battaglia M Passetti E Frey M. Occupational health and safety management in municipal waste companies: a note on the Italian sector. Saf Sci. (2015) 72:55–65. 10.1016/j.ssci.2014.08.002

[88] Zeng SX Shi JJ Lou GX. A synergetic model for implementing an integrated management system: an empirical study in China. J Clean Prod. (2007) 15:1760–7. 10.1016/j.jclepro.2006.03.007

[89] Micheli GJL Gnoni MG de Merich D Sala G Rosso A Tornese F. Barriers, drivers and impact of a simplified occupational safety and health management system in micro and small enterprises. In:Arezes P, editor. Advances in Intelligent Systems and Computing. Cham: Springer Verlag (2019). p. 81–90. 10.1007/978-3-319-94589-7_8

[90] Acakpovi A Dzamikumah L. An investigation of health and safety measures in a hydroelectric power plant. Saf Health Work. (2016) 7:331–9. 10.1016/j.shaw.2016.04.006 27924237 PMC5127901

[91] Rajaprasad SVS Chalapathi PV. Factors influencing implementation of OHSAS 18001 in Indian Construction Organizations: interpretive structural modeling approach. Saf Health Work. (2015) 6:200–5. 10.1016/j.shaw.2015.04.001 26929828 PMC4674494

[92] Bevilacqua M Ciarapica FE de Sanctis I. How to successfully implement OHSAS 18001: the Italian case. J Loss Prev Process Ind. (2016) 44:31–43. 10.1016/j.jlp.2016.08.004

[93] Rocha RS. Institutional effects on occupational health and safety management systems. Hum Factors Ergon Manuf Serv Ind. (2010) 20:211–25. 10.1002/hfm.20176

[94] Ramli AA Watada J Pedrycz W. Possibilistic regression analysis of influential factors for occupational health and safety management systems. Saf Sci. (2011) 49:1110–7. 10.1016/j.ssci.2011.02.014

[95] Zwetsloot GIJM Zwanikken S Hale A. Policy expectations and the use of market mechanisms for regulatory OSH certification and testing regimes. Saf Sci. (2011) 49:1007–13. 10.1016/j.ssci.2010.12.006

[96] Hale AR Swuste P. Safety rules: procedural freedom or action constraint? Saf Sci. (1998) 29:163–77. 10.1016/S0925-7535(98)00020-4

[97] Hollnagel E. Barriers and accident prevention. Hampshire: Ashgate (2004).

[98] Hale A Borys D Adams M. Safety regulation: the lessons of workplace safety rule management for managing the regulatory burden. Saf Sci. (2015) 71:112–22. 10.1016/j.ssci.2013.11.012

[99] Castillo C Shahriari M Casarejos F Arezes P. Prioritization of leading operational indicators in occupational safety and health. Inte J Occup Saf Ergon. (2022) 26:1–9. 10.1080/10803548.2022.2082693 35622396

[100] Podgórski D. Measuring operational performance of OSH management system - a demonstration of AHP-based selection of leading key performance indicators. Saf Sci. (2015) 73:146–66. 10.1016/j.ssci.2014.11.018

[101] Karanikas N Weber D Bruschi K Brown S. Identification of systems thinking aspects in ISO 45001:2018 on occupational health & safety management. Saf Sci. (2022) 148:105671. 10.1016/j.ssci.2022.105671

[102] Kano N Seraku N Takahashi F Tsuji S. Attractive quality and must-be quality. J Jpn Soc Qual Control. (1984) 14:147–56.

[103] Walters D Wadsworth E Marsh K Davies R Lloyd-Williams H. Worker representation and consultation on health and safety: an analysis of the findings of the European Survey of Enterprises on New and Emerging Risks (ESENER). (2012). Available online at: https://osha.europa.eu/en/publications/worker-representation-and-consultation-health-and-safety-analysis-findings-european (accessed December 22, 2023).

[104] Walters D Johnstone R Bluff E Jørgen Limborg H Gensby U. Improving compliance with occupational safety and health regulations: an overarching review. (2021). Available online at: https://osha.europa.eu/en/publications/improving-occupational-safety-and-health-changing-world-work-what-works-and-how (accessed December 22, 2023).

